# NMDA receptors in mouse anterior piriform cortex initialize early odor preference learning and L-type calcium channels engage for long-term memory

**DOI:** 10.1038/srep35256

**Published:** 2016-10-14

**Authors:** Bandhan Mukherjee, Qi Yuan

**Affiliations:** 1 Biomedical Sciences, Faculty of Medicine, Memorial University of Newfoundland, St. John’s, A1B 3V6, Canada

## Abstract

The interactions of L-type calcium channels (LTCCs) and NMDA receptors (NMDARs) in memories are poorly understood. Here we investigated the specific roles of anterior piriform cortex (aPC) LTCCs and NMDARs in early odor preference memory in mice. Using calcium imaging in aPC slices, LTCC activation was shown to be dependent on NMDAR activation. Either D-APV (NMDAR antagonist) or nifedipine (LTCC antagonist) reduced somatic calcium transients in pyramidal cells evoked by lateral olfactory tract stimulation. However, nifedipine did not further reduce calcium in the presence of D-APV. In mice that underwent early odor preference training, blocking NMDARs in the aPC prevented short-term (3 hr) and long-term (24 hr) odor preference memory, and both memories were rescued when BayK-8644 (LTCC agonist) was co-infused. However, activating LTCCs in the absence of NMDARs resulted in loss of discrimination between the conditioned odor and a similar odor mixture at 3 hr. Elevated synaptic AMPAR expression at 3 hr was prevented by D-APV infusion but restored when LTCCs were directly activated, mirroring the behavioral outcomes. Blocking LTCCs prevented 24 hr memory and spared 3 hr memory. These results suggest that NMDARs mediate stimulus-specific encoding of odor memory while LTCCs mediate intracellular signaling leading to long-term memory.

How synaptic signals are transmitted to the nucleus of the neuron to initiate the gene transcription required for long-term memory has been a topic of intense investigations. Calcium as a 2^nd^ messenger initiates cascades of intracellular signalling that are critically involved in synaptic plasticity and learning. Calcium-stimulated activation of cAMP response element binding protein (CREB) and CRE-mediated gene transcription are a universal requirement in memory formation across species^1^. Voltage-gated calcium channels such as the L-type calcium channels (LTCCs) and NMDA receptors (NMDARs) serve as the principal sites for calcium entry at the membrane, and are responsible for the activation of altered gene expression^2^,^3^. While both channels are involved in synaptic plasticity mechanisms such as long-term potentiation (LTP), a putative cellular mechanism for memory formation, they differ in their roles in LTP induction and intracellular signalling^4^,^5^,^6^,^7^,^8^.

NMDARs have been regarded as co-incident detectors for presynaptic activity and postsynaptic depolarization during LTP induction^9^, which permits calcium entry at the synaptic site^10^. LTCCs, which are localized in the somatic membrane and proximal dendrites^11^,^12^,^13^,^14^, have an important role in translating cytosolic calcium increases to gene expression changes^15^,^16^,^17^,^18^,^19^. In the hippocampus, both NMDAR-dependent, and LTCC-dependent LTP has been reported in rodents^4^,^5^,^6^,^7^,^20^ as well as in humans^8^. Depending on the induction protocols, short-duration LTP (lasting less than 1 hr) can be induced by a single high frequency or theta burst stimulation, which is abolished by NMDAR blockers and does not require CRE-mediated transcription. Longer-duration LTP (lasting hours) requires stronger induction (e.g. multiple theta bursts or high frequency trains) and is dependent on LTCC and CRE-mediated transcription^16^,^21^. These two calcium channels are also involved in LTP in various other structures including the amygdala^22^,^23^, anterior cingulate gyrus^24^, insular cortex^25^, superior colliculi^26^, and olfactory bulb^27^. Concurrently, both NMDARs and LTCCs are implicated in various learning models such as spatial memory^28^,^29^, fear conditioning^22^ and associative olfactory learning^11^,^27^.

Early odor preference learning can be induced in neonatal rat^30^,^31^ or mouse^32^,^33^ by pairing a novel odor with a tactile stimulus that signals maternal care (e.g. stroking the body of the pup with a brush). This model has the advantage of being well-defined with respect to the sites of learning and the temporal phases of the memory (short-term memory (STM) *vs.* long-term memory (LTM))^34^, therefore it is an ideal model to study memory mechanisms. The piriform cortex is critically involved in odor memory encoding. Blocking NMDARs in the anterior piriform cortex (aPC) prevents odor preference learning in pups and LTP induction *in vitro*^35^. However, whether LTCCs in the aPC are necessary for early odor learning has not been tested. In this study we first investigated the relationship of the NMDARs and LTCCs in generating somatic calcium transients in aPC pyramidal neurons, and then we studied the interaction of the NMDARs and LTCCs in odor preference learning in week-old neonatal mice.

## Materials and Methods

### Subjects

Postnatal day (PD) 7–9 C57BL/6 mouse pups (Charles River) of both sexes were subjects. Mice were bred on site and housed under a 12 h light/dark cycle with *ad libitum* dry food and water. Procedures were consistent with Canadian Council of Animal Care guidelines, and approved by the Memorial University Institutional Animal Care Committee.

### Fluorescence Immunohistochemistry

PD 8–10 pups were anaesthetized with pentobarbital i.p. (150 mg/kg, Rafter 8 Products) and perfused transcardially with saline (0.9%), followed by paraformaldehyde (4%, dissolved in 0.1 M PBS). Brains were collected and placed in 4% paraformaldehyde overnight at 4 °C, and then transferred to a sucrose solution (20%) for an additional 24 h before slicing.

For slicing, 25 μm coronal sections were cut using a cryostat (HM550, Thermo Scientific) and mounted on chrome-gelatin coated slides. Slides were kept at 4 °C for 10 min before being brought to room temperature to dry. A LTCC anti-Cav1.2 antibody (1:200, Alomone Labs) was applied to the slides. The antibody was dissolved in phosphate buffered saline (PBS) with 0.2% Triton-X-100, 0.02% sodium azide, and 5% normal goat serum and left on sections overnight at 4 °C in a humidified chamber. The following day, the slides were washed with PBS and a goat anti-rabbit Alexa 488 2^nd^ antibody (1:200, Molecular Probe) was applied to the slices for 1 hr. Slides were then washed 3 × 10 min in PBS and coverslipped with anti-fade mounting medium (Vectashield, Vector). Images were taken with a Fluoview FV1000 confocal microscope (Olympus) and processed in Corel Photo-Paint X4 software.

### Calcium Imaging

Pups were decapitated under halothane anesthesia. Sagittal PC slices (300 μm) were cut in an ice-cold sucrose cutting solution (in mM: 83 NaCl, 2.5 KCl, 3.3 MgSO_4_, 1 NaH_2_PO_4_, 26.2 NaHCO_3_, 22 glucose, 72 sucrose, 0.5 CaCl_2_) equilibrated with 95% O_2_/5% CO_2_^36^,^37^. Slices were then incubated in the same sucrose solution containing Oregon Green BAPTA-1 AM (10–20 μM, with 0.02% Pluronic F-127, Molecular Probes) at 34 °C for 30–60 min, before washed and left in no-dye solution at room temperature. Recording was conducted in an open bath chamber where slices were perfused with artificial cerebrospinal fluid (aCSF in mM: 110 NaCl, 2.5 KCl, 1.3 MgSO_4_, 1 NaH_2_PO_4_, 26.2 NaHCO_3_, 22 glucose, 2.5 CaCl_2_) at 30.0 °C. Slices were visualized with an Olympus BX51WI upright microscope.

*In vitro* population calcium imaging followed established procedures^36^. Image acquisition (488 nm excitation, 2 × 2 binning, 15 Hz) was carried out with a cooled-CCD camera system (Andor Clara, T.I.L.L. Photonics). A concentric bipolar stimulating electrode (FHC) was placed in the lateral olfactory tract (LOT) of the aPC. LOT was stimulated (25–30 μA) by an ISO-Flex stimulator (AMPI) with a 200 μsec pulse. Sagittal slice cutting and recording configurations are demonstrated in Supplementary Figure 1. Drugs used included APV (50 μM, Sigma Aldrich), nifedipine (10 μM, Tocris), BayK 8644 (20 μM, Tocris) and NBQX (40 μM; Tocris).

**Figure 1:**
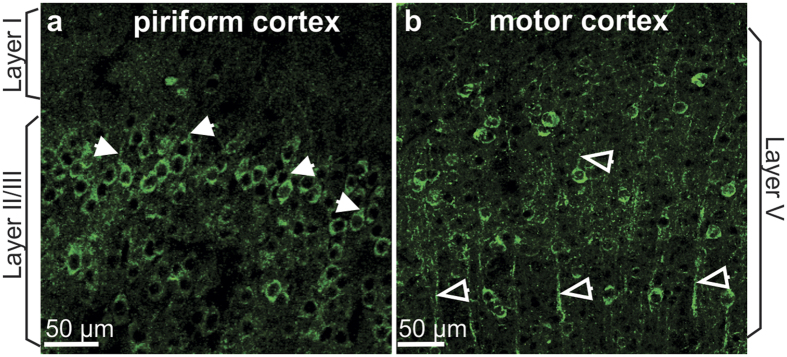
L-type calcium channels (LTCCs) are expressed in the piriform cortex pyramidal cells. (**a**) LTCC expression in the anterior piriform cortex layer II/III pyramidal cells using an antibody against Cav1.2 channels. Solid arrows indicate the LTCC staining at the base of the apical dendrites. (**b**) LTCC expression in the motor cortex layer V pyramidal cells. Note that apical dendritic shafts (arrows) are stained.

Image processing and analysis were performed with ImageJ (NIH) and Excel. Activity maps of cell ensembles were constructed by averaging 4–6 frames of evoked calcium responses from 5 stimulus trials, background subtracted and median filtered for illustration only (examples in Figs 1a1,b1, 2a1,b1 and 3a1,b1). Changes of somatic [Ca^2+^] were expressed as relative fluorescence changes (ΔF/F, where F is the baseline fluorescence before a stimulus and ΔF is the evoked change in fluorescence). ΔF/F was measured in areas of interest in the soma of layer II/III pyramidal cells. The average of the three peak frames immediately following the stimulation was used to indicate the size of the calcium transient. Fifteen to thirty-five cells were randomly selected in each slice imaged.

### Behavioral Studies

Behavioral experiments were carried out in a temperature-controlled room (27 °C) and followed previously established protocols^32^,^35^ as described below.

#### Drug infusion

Intracerebral drug infusions were carried out on PD7 pups following cannula surgeries. Pups were anesthetized *via* hypothermia (under ice) and placed skull flat in a stereotaxic apparatus. An incision of the skin was made to expose the skull where two small holes were drilled. Two infusion cannulas (Vita Needle, MA) were inserted into the brain at specific coordinates for aPC (1.8 mm anterior and 2 mm bilateral, 3.5 mm ventral with respect to bregma). The aPC coordinates were verified with 4% methylene blue dye or fluorescence bead (example see Supplementary Figure 2) in pilot experiments (n = 6). Drugs or vehicle were infused directly via the cannulas. Half μl of the desired solution was infused bilaterally at a rate of 0.25 μl/min using a Hamilton syringe operated by a precision pump (Fusion 400, Chemyx Inc). The infusion tubing and cannula was left for another min before being withdrawn gently from the brain and the skin was sutured. The pups were allowed to recover on the warm bedding for 30 min before odor training. Pharmacological agents used included D-APV (500 μM, dissolved in saline), nifedipine (100 μM, dissolved in 1% ethanol + saline), and a cocktail of D-APV (500 μM) and BayK 8644 (200 μM, dissolved in 1% ethanol + saline). The vehicle used was 1% ethanol + saline.

#### Odor preference training

After drug or vehicle infusion, pups were subjected to an odor plus stroking (O/S^+^) or an odor only (O/S^−^) condition. Pups in the O/S^+^ groups were placed on peppermint-scented bedding (0.3 ml peppermint extract in 500 ml bedding) and stroked with a paintbrush at 30 sec interval for 10 min, thus comprising 10 trials of 30 sec stroking, interleaved by 30 sec rests. Pups in the O/S^−^ groups were placed in peppermint-scented bedding for 10 min without being stroked. All pups were returned to the dam after training.

#### Odor preference testing

Three or twenty-four hours following the odor training, pups were tested for odor preference memory in a testing apparatus. The apparatus contained a stainless steel box (30 × 20 × 18 cm) placed over two training boxes separated by 2 cm. One box contained peppermint-scented bedding and the other contained unscented bedding, or vanillin-scented bedding (0.3 ml vanillin in 500 ml bedding; dissimilar odor test), or peppermint (70%) +vanillin (30%) mixture bedding (similar odor test). During testing, pups were placed in the 2 cm central zone. Times that pups spent over peppermint-scented versus other bedding were recorded in five one-minute trials. Pups were allowed 1 min rest between the trials in a clean cage. The percentage of the time spent over peppermint bedding over total time spent over either bedding was calculated for each pup. To evaluate stimulus specific memory, rats were tested alternately with dissimilar odors or similar odors first, followed with the other odor pairs 10 min later.

### Synaptic AMPAR measurement

In separate cohorts, 3 hr following odor training, pups were decapitated, and aPCs were collected and flash frozen on dry ice^38^. The aPC is located posterior to the olfactory bulbs but anterior to the termination of the LOT. The LOT is visible on the ventral surface of the brain and the aPC lies dorsal and lateral to it. Tissue was collected in a triangular shape from the ventral surface of the brain at and lateral to the LOT, posterior to the olfactory bulbs, and anterior to the termination of the LOT. Samples were stored at −80 °C until further processing.

#### Synaptic membrane isolation

Extraction of synaptic membrane followed previously published procedures^38^. Tissue samples were homogenized in sucrose buffer (300 μl) on ice containing (in mM): 320 sucrose, 10 Tris (pH7.4), 1 EDTA, 1 EGTA, 1X complete protease inhibitor mixture and phosphatase inhibitor mixture (Roche). The homogenized tissue was centrifuged at 1000 rpm for 10 min. The supernatant was taken and centrifuged at 10,000 rpm for 30 min to obtain a pellet, which was subsequently re-suspended in 120 μl sucrose buffer using a pestle mixing/grinding rod (Thomas Scientific) directly in the microfuge tube. Eight volumes of Triton X-100 buffer (in mM: 10 Triton-100, 1 EDTA, 1 EGTA, 1X protease and phosphatase inhibitors pH7.4) were added for detergent extraction (final 0.5% v/v). This suspension was incubated at 4 °C for 35 min with gentle rotation and then centrifuged at 28,000 rpm for 30 min. The pellet containing postsynaptic densities and synaptic junctions that are insoluble in Triton X-100^39^ was re-suspended in 100 μl of TE buffer (in mM: 100 Tris, 10 EDTA, 1% SDS, 1X protease and phosphatase inhibitors), sonicated, boiled for 5 min and stored at −80 °C until use. Protein concentrations for each sample were determined by a BCA protein assay kit (Pierce). The volume of lysate required to make 35 μg of protein for each sample was calculated.

#### Western blotting

A total of 100 μl lysate solution, sample buffer (0.3 M Tris-HCl, 10% SDS, 50% glycerol, 0.25% bromophenol blue, 0.5 M dithiothreitol), and dH2O were prepared and boiled for 2 min at 100 °C. Samples and a protein ladder (Thermo Scientific) were loaded into a 7.5% SDS-PAGE gel. Sample separation by SDS-PAGE was followed by transference to a nitrocellulose membrane (Millipore). Membranes were cut horizontally at the 72 kDa level, and the upper portion was probed with a rabbit antibody for GluA1 subunits (1:7000, Cell Signalling)^40^, and the lower portion was probed for β-actin (1:5000, Cell Signalling). Membranes were incubated in primary antibody overnight at 4 °C on a shaker. Next day membranes were washed 3 × 5 min with 1X TBST. HRP-bounded 2^nd^ antibodies were applied (1:10,000, anti-rabbit; Pierce) for 1 hr. Membranes were then washed 3 × 10 min in TBST and enhanced chemiluminescence Western blotting substrate (Pierce) was applied. Blots were then developed on x-ray film (AGFA). Films were scanned using an image scanner (CanoScan LiDE 200), and the optical density (OD) of each band was measured using ImageJ software. Each sample was normalized to the corresponding β-actin band that was run on the same gel.

#### Statistical Analyses

Statistical analyses were performed using OriginPro software (Originlab, MA). Data were presented as Mean ± S.E.M. One-way ANOVAs were used for behavioural tests with Fischer LSD *post-hoc* comparisons to evaluate differences between behavioural groups. One-way ANOVAs with Fisher LSD *post-hoc* tests or paired t-tests were used for Western blotting and calcium imaging data.

## Results

### LTCC activation is dependent on NMDAR activation in aPC pyramidal cells

We first looked at the LTCC Cav1.2 expression in the aPC using immunohistochemistry. LTCCs were expressed in the membrane of the soma and the base of the apical dendrites of pyramidal cells in layer II/III (Fig. 1a, n = 3), similar to the expression pattern in the hippocampus as reported previously^14^. In contrast, in neocortex such as the motor cortex, LTCCs were also expressed in the shaft of apical dendrites (Fig. 1b). In neither area did we observe LTCC expression in distal dendritic arbors as is normally observed for NMDARs^41^,^42^. However, we cannot exclude the possibility that LTCCs are expressed in distal dendrites in low density that is beyond the detection threshold in our method.

**Figure 2:**
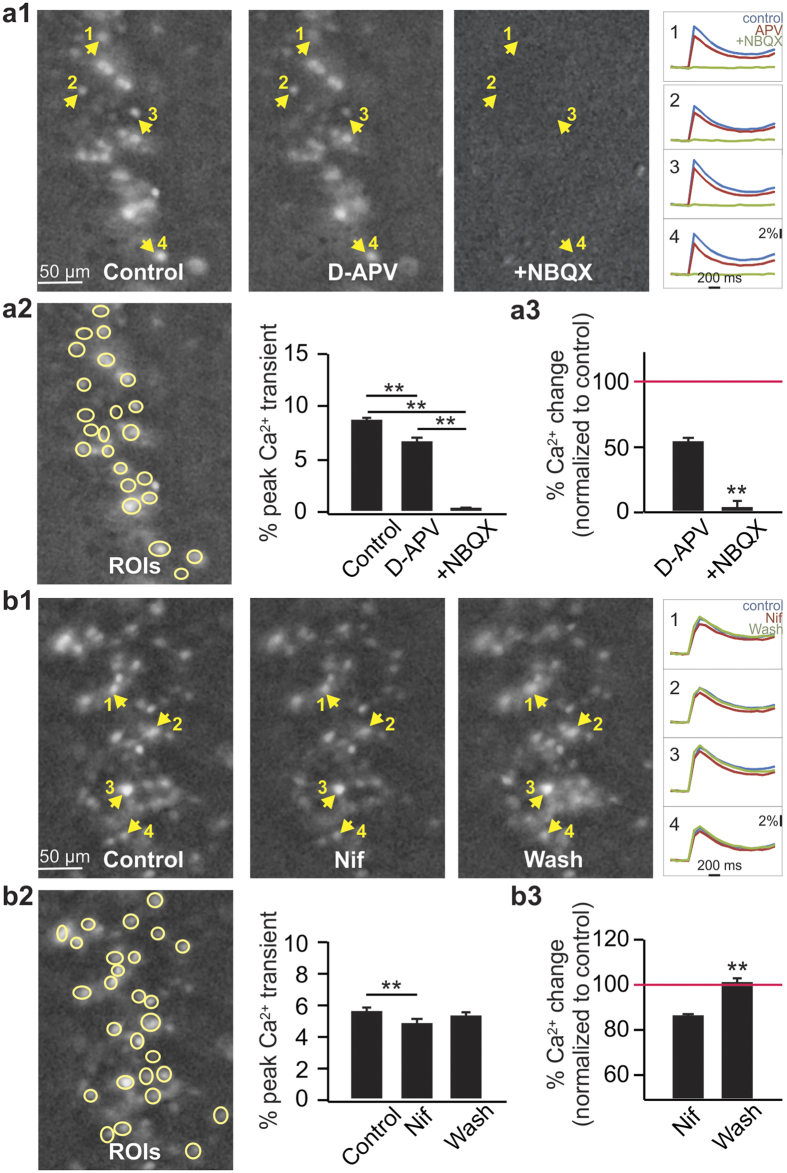
Lateral olfactory tract (LOT) stimulation activates LTCCs. (**a1–a3**) Somatic calcium transients in the anterior piriform pyramidal cells by LOT stimulations are dependent on postsynaptic AMPARs and NMDARs. **(a1**) Example images of population calcium imaging evoked by a single LOT stimulation in control, D-APV and D-APV+ NBQX conditions. Images were constructed by averaging 4–6 frames of evoked calcium responses (ΔF/F) from 5 stimulus trials. Example calcium transient traces from 4 cells are shown on the right. (**a2**) Peak calcium transients (ΔF/F%) averaged from a population of cells (yellow circles, regions of interest) on the same slice. (**a3**). Normalized calcium changes (to control) during D-APV and D-APV+ NBQX applications from 4 slices (n = 80 cells). (**b1–b3**) Blockade of LTCCs reduce somatic calcium transients in pyramidal cells. (**b1**) Example images of population calcium imaging in control, nifedipine (Nif) and Nif washout conditions. (**b2**). Peak calcium transients averaged from a population of cells on the same slice. (**b3**) Normalized calcium changes during Nif and Nif washout from 4 slices (n = 110 cells). **p < 0.01.

We then studied the effects of LTCC or NMDAR blockade on somatic calcium transient evoked by LOT stimulation. Action potentials in dye-loaded cells elicit somatic calcium transients such that cells recruited by LOT stimulation can be identified^36^,^43^. Cells with somatic transients were largely confined to the pyramidal cell layers. Similar to evoked EPSCs, somatic calcium transients correlates positively with LOT stimulation intensities (Supplementary Figure 3). The somatic calcium transient was reduced in the presence of APV and abolished when NBQX was added (Fig. 2a1, 2, 3), suggesting calcium transients seen here were post-synaptic responses evoked by the LOT stimulation. With the moderate stimulation intensities used (25–30 μA), single LOT stimulation evoked ~2–10% somatic calcium increase in individual cells (e.g. Figs 2a2,b2 and 3a2b2). On average, APV reduced single LOT stimulation evoked calcium transient to 54.2 ± 2.7% of the baseline, while the residual calcium was almost abolished (4.2 ± 3.1% of the baseline) in the presence of NBQX (n = 80 cells from 4 slices, t = 12.38, p < 0.001 compared to APV; Fig. 2a3). Adding nifedipine to aCSF reduced calcium transients to 86.3 ± 0.8% of the baseline, which was reversed following 30 min wash (101.6 ± 0.02%, n = 110 cells from 4 slices, t = 10.61, p < 0.001 compared to nifedipine, Fig. 2b1, 2, 3).

**Figure 3:**
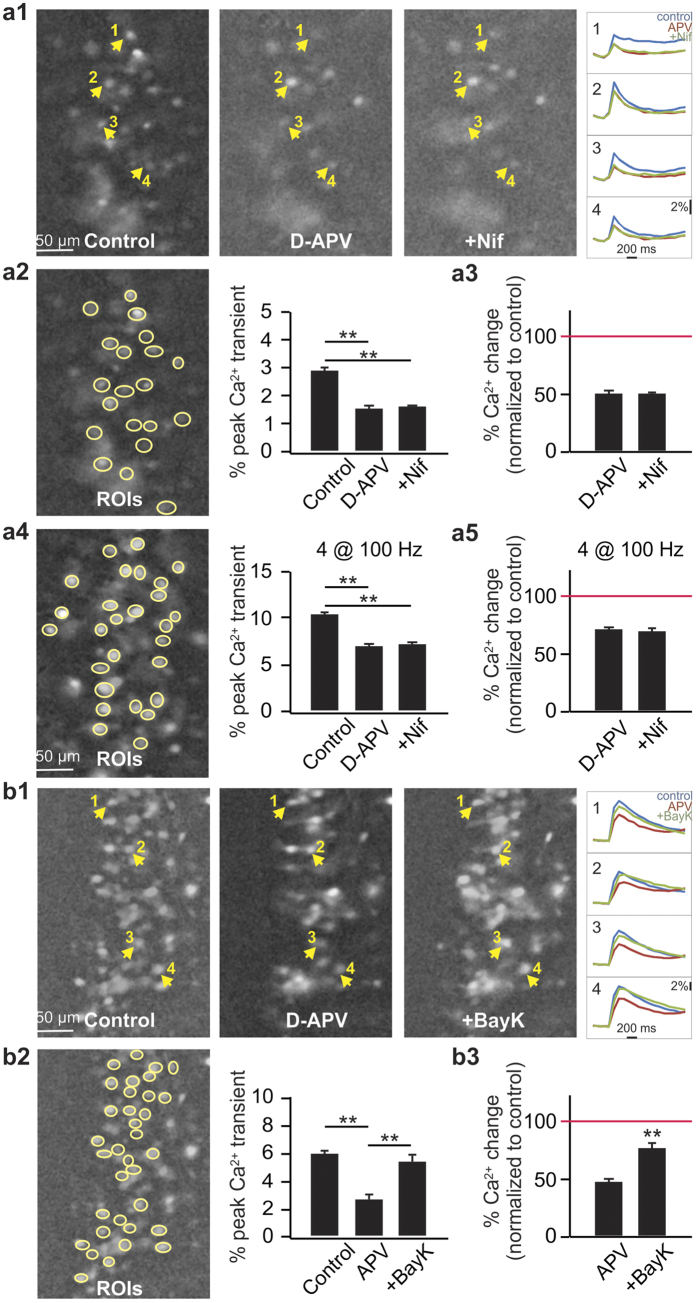
LTCC activation is subsequent to NMDAR activation. (**a1–a5**) Nifedipine does not further reduce calcium transients in the presence of NMDAR blockade by D-APV. (**a1**) Example images of population calcium imaging in control, D-APV and D-APV + Nif conditions. Example calcium transient traces from 4 cells are shown on the right. (**a2**) Peak calcium transients (ΔF/F%) to a single LOT stimulation, averaged from a population of cells (yellow circles) on the same slice. (**a3**) Normalized calcium changes (to control) to a single LOT stimulation during D-APV and D-APV + Nif applications from 3 slices (n = 55 cells). (**a4**) Peak calcium transients to 4 LOT stimulations at 100 Hz. (**a5**) Normalized calcium changes to 4 LOT stimulations from 3 slices (n = 70 cells). (**b1–b3**) Application of BayK-8644 increased somatic calcium transients in the presence of D-APV. (**b1**) Example images of population calcium imaging in control, D-APV and D-APV + BayK-8644 conditions. (**b2**) Peak calcium transients averaged from a population of cells on the same slice. (**b3**) Normalized calcium changes during D-APV and D-APV + BayK-8644 from 5 slices (n = 125 cells). **p < 0.01.

We next studied the interaction of the LTCC and NMDAR in eliciting somatic calcium transients. In the presence of APV, nifedipine failed to further reduce the somatic calcium transient (50.7 ± 0.02% of the baseline in APV *vs.* 50.0 ± 0.02% in APV + nifedipine, t = 0.68, p = 0.49, n = 55 cells from 3 slices; Fig. 3a1, 2, 3). This suggests that LTCC activation was subsequent to the NMDAR activation. We then tested whether a stronger stimulus could recruit LTCCs directly as may happen during theta burst or high frequency stimulation. When 4 LOT stimulations at 100 Hz were used, nifedipine still failed to further reduce calcium transients (70.8 ± 0.01% in APV vs. 70.0 ± 0.01% in APV+ nifedipine, t = 1.13, p = 0.26, n = 70 cells from 3 slices; Figs 3a4 and 5). It is noted that the calcium transient evoked with the train stimulation has a smaller NMDAR component (30%) compared to that in the single stimulus (50%), suggesting additional recruitment of other voltage-gated calcium channels or mGluRs under the stronger stimulation.

If LTCCs act downstream of the NMDARs, then direct activation of the LTCCs in the presence of NMDAR blockade should allow additional calcium influx to the cells. This is indeed the case when BayK 8644, a LTCC agonist was added in the presence of APV. Single LOT stimulation was used. BayK 8644 increased the calcium transient to 76.0 ± 4.4% compared to 46.9 ± 2.9% in APV only (t = 11.53, p < 0.001, n = 125 cells from 5 slices; Fig. 3b1, 2, 3).

### Differential roles of the NMDAR and LTCC in early odor preference learning

**Figure 4:**
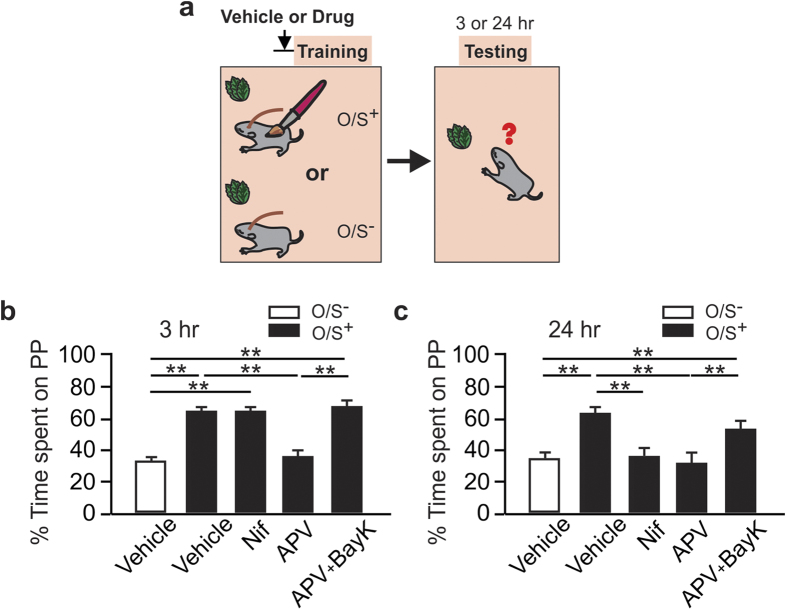
Differential roles of NMDARs and LTCCs in early odor preference learning. (**a**) Schematics of the odor preference training and testing paradigm. (**b**) Percentage of time spent over peppermint (PP)-scented bedding at 3 hr testing. (**c**) Percentage of time spent over PP-scented bedding at 24 hr testing. **p < 0.01.

We then pursued behavioral experiments to test the roles of the NMDAR and LTCC in early odor preference learning in neonate mice. We tested the effects of NMDAR or LTCC blockade on short-term (3 hr) and long-term (24 hr) memory^44^ (Fig. 4a), and tested whether BayK 8644 could rescue the learning from NMDAR blockade at the two time points.

NMDAR blockade prevented 3 hr memory while blocking LTCC had no effect on this short-term memory. One-way ANOVA shows significant differences in treatment conditions (F_4,20_ = 53.66, p < 0.001; Fig. 4b). *Post-hoc* Fisher test shows a significant difference between the O/S^+^ + vehicle (65.18 ± 1.78) and the O/S^−^ + vehicle (33.09 ± 2.06) groups (n = 5, t = 9.70, p < 0.001). D-APV infusion prevented odor preference memory (36.83 ± 2.68%, n = 5, t = 8.57, p < 0.001) while the nifedipine group showed comparable odor preference (65.11 ± 1.56%) compared to the O/S^+^ + vehicle group (t = 0.02, p > 0.05). Activating LTCCs with BayK-8644 rescued the 3 hr odor preference memory from the NMDAR blockade (67.92 ± 3.22, n = 5, t = 9.40, p < 0.01 compared to the D-APV only group).

For the 24 hr memory, both NMDAR and LTCC blockade prevented it. One-way ANOVA shows significant group effects (F_4,30_ = 8.69, p < 0.001; Fig. 4c). *Post-hoc* Fischer test shows a significant difference between the O/S^+^ + vehicle (62.87 ± 3.92, n = 7) and the O/S^−^ + vehicle (35.08 ± 2.93) groups (n = 7, t = 4.29, p < 0.01). Both nifedipine (35.61 ± 5.31, n = 6) and APV (32.56 ± 5.32, n = 7) prevented 24 hr preference memory compared to the O/S^+^ + vehicle pups (p < 0.001). However, adding BayK 8644 to the APV rescued the learning at 24 hr (53.54 ± 4.95, n = 8, t = 3.34, p < 0.01) compared to the D-APV only group.

### NMDAR blockade impairs stimulus-specific discrimination of the conditioned odor

**Figure 5:**
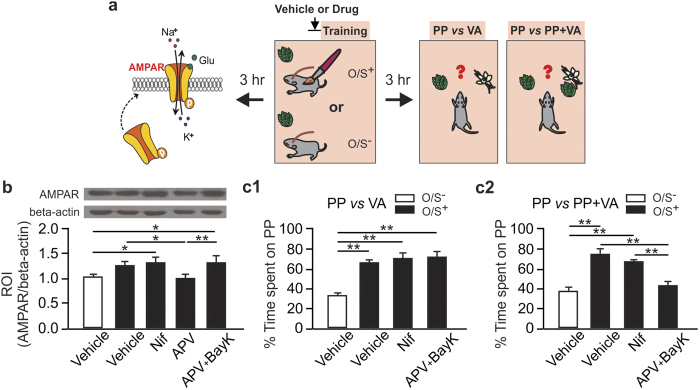
NMDARs but not LTCCs mediates input-specific discrimination of the conditioned odor. (**a**) Schematics of the odor preference training and testing paradigm. (**b**) Relative optical densities (ROIs) of AMPAR expression (normalized to beta-actin) in various groups at 3 hr post training. Full-length blots are presented in Supplementary Figure 2. (**c1**) Percentage of time spent over peppermint (PP)-scented bedding when tested with two dissimilar odors. (**c2**) Percentage of time spent over PP-scented bedding when tested with PP *vs.* PP + VA (vanillin) mixture. *p < 0.05, **p < 0.01.

We were intrigued that either isolated NMDAR activation (in the presence of nifedipine) or isolated activation of LTCCs (D-APV + BayK 8644) was able to induce 3 hr memory. AMPAR synaptic insertion is implicated in both short-term and long-term odor preference memory^40^,^45^. We tested the amount of AMPAR synaptic membrane expressions in these conditions (Fig. 5a,b; Full length blots are presented in Supplementary Figure 4). AMPAR synaptic expression at 3 hr in the aPC mirrored the behavioral outputs (F_4,24_ = 3.21, p < 0.05; Fig. 5b). The nifedipine group showed higher AMPAR expression (1.31 ± 0.10, n = 6) compared to the O/S^−^ + vehicle group (1.04 ± 0.04, n = 5, t = 2.12, p = 0.04). D-APV only prevented the AMPAR increase (1.00 ± 0.09, n = 6, t = 0.30, p = 0.77). Co-infusion of BayK-8644 increased AMPAR (1.34 ± 0.10, n = 6, t = 3.24, p < 0.01 compared to D-APV only group).

To understand whether and how the memories formed through either LTCCs or NMDARs differ, we performed experiments to test stimulus specificity of the 3 hr memory using odor discrimination between the conditioned odor peppermint, and a dissimilar odor vanillin, or an odor mixture (70% peppermint + 30% vanillin) (Fig. 5a). Dissimilar odor testing yielded peppermint preference patterns similar to that in the 3 hr peppermint *vs.* normal bedding test (F_3,25_ = 47.53, p < 0.001; Fig. 5c1). Both nifedipine (70.97 ± 3.83, n = 7) and D-APV + BayK 8644 (74.11 ± 2.45, n = 8) groups showed significantly higher time spent over peppermint bedding compared to the O/S^−^ + vehicle group (32.67 ± 2.50, n = 7, p < 0.01). It has been reported previously that rat pups form a generalized avoidance or approach to other novel odors when olfactory bulb GABAa receptor^46^ or CaMKII^40^ is blocked. To test whether aPC LTCC activation alone without NMDARs results in generalized approach response, we tested vanillin preference in a cohort of mouse pups. D-APV + BayK 8644 pups trained with peppermint did not show any preference to the novel odor vanillin (Supplementary Figure 5).

However, when peppermint was tested against a similar odor mixture (F_3,25_ = 35.93, p < 0.001; Fig. 5c2), the D-APV + BayK 8644 group showed no preference for the peppermint bedding (43.35 ± 2.94, n = 7) compared to the O/S^−^ + vehicle group (38.23 ± 2.99, n = 7, t = 1.22, p = 0.23), while the nifedipine group still showed a clear preference (67.26 ± 1.69, n = 7, t = 6.73, p < 0.01). The fact that in the D-APV + BayK 8644 group, the same pups showed preference for peppermint in the dissimilar odor test but no preference for peppermint in the similar odor test suggests that activating LTCCs alone in the absence of NMDAR activation results in loss of stimulus specificity of the odor memory–the memory is extended to other similar stimulus that has a large component overlapping with the conditioned stimulus.

## Discussion

NMDARs and LTCCs demonstrated differential roles in early odor preference learning in mouse pups. Previous work defined three temporal phases for early odor preference memory: a short-term memory (up to 3 hr) which is independent of transcription and translation, an intermediate memory (5 hr) which requires transcription but not translation, and a long-term memory (24 hr) which is dependent on both transcription and translation^47^. Blocking NMDARs during learning prevented both short-term (3 hr) and long-term (24 hr) memories. However, LTCC blockade prevented 24 hr memory but did not interrupt short-term memory. It is striking that LTCC blockade itself did not affect 3 hr memory and associated AMPAR increase, but activating LTCCs when the NMDARs were blocked nevertheless induced 3 hr memory and AMPAR increases. These results suggest that NMDARs, but not LTCCs, are normally required for 3 hr memory. However, when LTCCs are overdriven, it could compensate NMDAR loss to promote the AMPAR insertions needed for short-term memory.

The NMDAR and LTCC are involved in different forms of LTPs in hippocampus^16^,^21^, however, whether they engage in different phases of memory has not been tested. In amygdala, both NMDARs and LTCCs are required in LTPs with distinct induction protocols. Spike-timing dependent LTP generated by associating pre- and postsynaptic activities requires LTCCs but not NMDARs, while a tetanic stimulation engages NMDARs exclusively^22^. Interestingly, similar to what we observed in this study, NMDAR blockade prevents both short and long-term fear memory while blocking LTCCs exclusively affects only long-term memory^22^. However, how these two channels are differentially engaged in the signalling pathways that lead to either short-term or long-term memory is not known. Both long-term fear memory^48^ and odor preference memory^30^,^35^,^49^ require CREB signaling. Short-term memory may only require local AMPAR insertion into the synaptic membrane mediated by calcium activated CaMKII signalling^40^.

LTCCs are expressed in the somatic membrane and at the base of the apical dendrites of pyramidal cells in the aPC. This subcellular distribution of LTCCs in pyramidal cells in the aPC is consistent with that in other structures^11^,^12^,^13^,^14^ and implies a differential role of LTCCs in intracellular signalling from NMDARs. Early experiments in striatal neurons demonstrated sequential activation of AMPARs, NMDARs and LTCCs^18^. A model was put forward to suggest that LTCC activation is dependent on NMDARs due to their longer opening kinetics than those of AMPARs. Once activated, LTCC allows Ca^2+^ influx and activation of a kinase pathway to translocate to the nucleus to phosphorylate CREB at Ser133^18^. Our calcium imaging data showing NMDAR-dependent activation of the LTCC is consistent with this model.

Detailed downstream signalling from these calcium channels is best characterized in the hippocampal neurons. In the hippocampus, although both the NMDAR and LTCC are driven by physiologically relevant synaptic inputs to engage CREB signaling^15^, the LTCC appears to be particularly important in coupling synaptic signalling to the nucleus^15^,^50^. α_1c_-comprised LTCC contains a calmodulin (CaM) binding domain and calcium influx through LTCC activates CaM, leading to phosphorylation of CREB^50^. Various routes are suggested to mediate CaM signalling from the cytosol to the nucleus^51^. Either CaM translocates to the nucleus to activate calmodulin kinase IV (CaMKIV)^15^,^17^,^52^, or it activates other kinases (e.g. mitogen-activated protein kinase, MAPK), which in turn translocate to the nucleus to phosphorylate CREB^50^.

Another intriguing finding is that LTCC activation in the absence of NMDAR activation results in impaired discrimination of the conditioned odor from a similar odor mixture. This suggests a critical role of NMDAR in mediating the stimulus specificity in early odor preference learning. NMDAR hypofunction has been linked to impaired pattern separation in the hippocampal dentate gyrus^53^. We propose that synaptic NMDARs associate odor-induced glutamate input with stroking/norepinephrine induced excitation of pyramidal cells to initiate memory encoding and ensure input-specificity of the learning by activating CaMKII signalling and CaMKII- mediated AMPAR insertion^40^. Meanwhile, the activation of the NMDAR leads to prolonged depolarization of the pyramidal cells and subsequently engages LTCCs. Calcium influx through LTCCs initiates CaM-mediated protein kinase translocation into the nucleus to activate CREB transcription. Direct activation of LTCCs without NMDARs may lead to AMPA insertion that affects a broader range of synapses.

Interestingly, cognitive decline during aging has been associated with increased LTCC activities in the hippocampus^54^,^55^,^56^. There is a shift from NMDAR-dependent LTP to LTCC-dependent LTP in the aging hippocampus^57^,^58^. Abnormal activity of hippocampal neurons are correlated with impaired pattern separation ability both in aged humans^59^ and in aged animals^60^. Olfaction dysfunction is also common in aging populations and is one of the earliest signs indicating Alzheimer’s disease (AD) development^61^,^62^. However, it is not known whether altered expressions and functions of LTCCs in the aPC underlie the olfactory deficiency in AD patients.

In summary, our results highlight the importance of balanced NMDAR and LTCC functions in encoding input-specific long-term memory.

## Additional Information

**How to cite this article**: Mukherjee, B. and Yuan, Q. NMDA receptors in mouse anterior piriform cortex initialize early odor preference learning and L-type calcium channels engage for long-term memory. *Sci. Rep.* **6**, 35256; doi: 10.1038/srep35256 (2016).

## Supplementary Material

Supplementary Information
