# Supplementary material for: NMDA receptors in mouse anterior piriform cortex initialize early odor preference learning and L-type calcium channels engage for long-term memory
Source: Sci Rep. 2016 Oct 14;6:35256. doi: 10.1038/srep35256 (PMC5064360; doi:10.1038/srep35256)
Supplement: Supplementary Information [file srep35256-s1.pdf]

Supplementary Figures

NMDA receptors in mouse anterior piriform cortex initialize early odor preference learning and L-type calcium channels engage for long-term memory

Bandhan Mukherjee[1], Qi Yuan[1*]

[1]Biomedical Sciences, Faculty of Medicine, Memorial University of Newfoundland, St. John's, A1B 3V6, Canada

***Corresponding author:** qi.yuan@med.mun.ca

Supplementary Figure 1

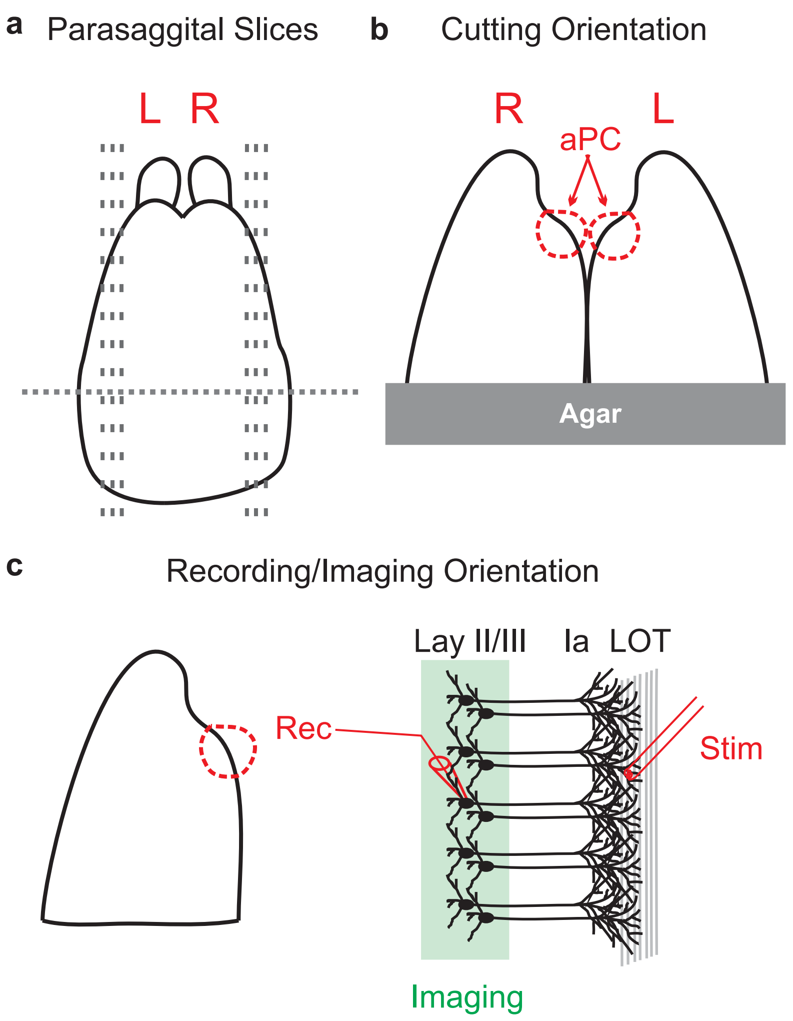

Tissue preparations for electrophysiological and imaging analysis

(a) Dorsal view of the whole brain. Dashed lines represent sagittal slice locations. (b) View of tissue after mid sagittal cut. Hemispheres are resting with the mid sagittal suture facing down with the olfactory bulbs facing inward. Dashed circles indicate approximate location of the anterior piriform cortex (aPC). (c) View of slices in the recording chamber and under the microscope indicating electrode placements and area of interest for imaging (green, layer II/III). Dashed circle represents the approximate location of the aPC.

Supplementary Figure 2

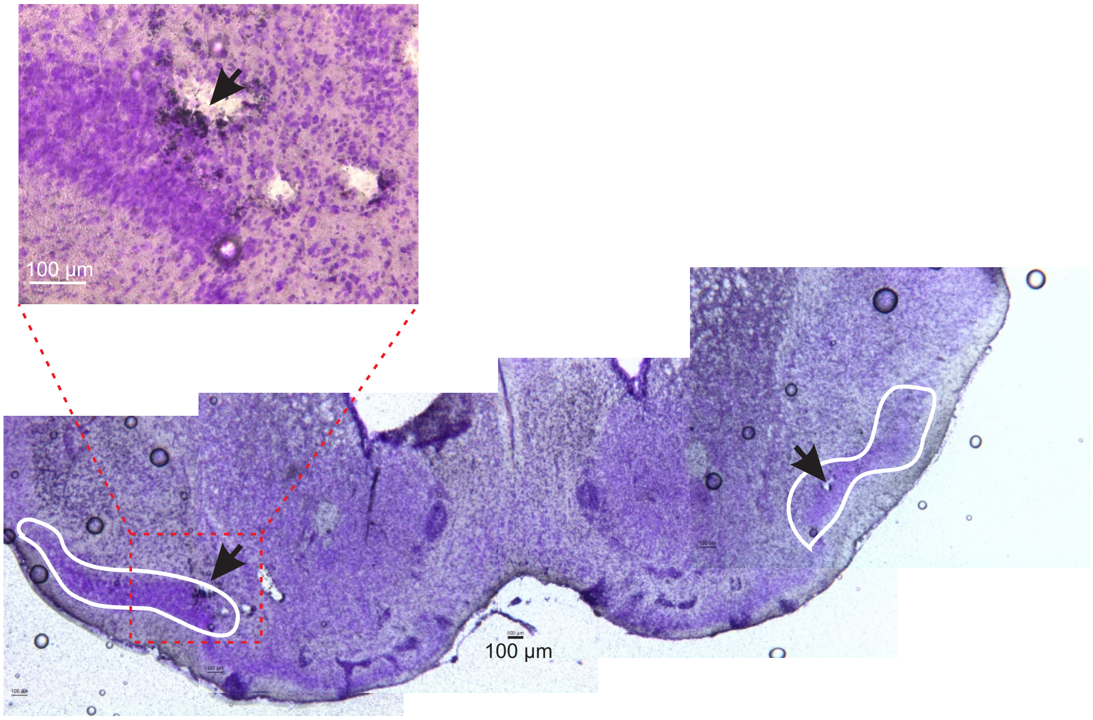

Example injection site in the anterior piriform cortex using fluorescence bead

Nissl staining of a fluorescence bead injected brain. Black arrows indicate the injected beads (black with the Nissl staining) within the anterior piriform cortex (aPC, white traces).

Supplementary Figure 3

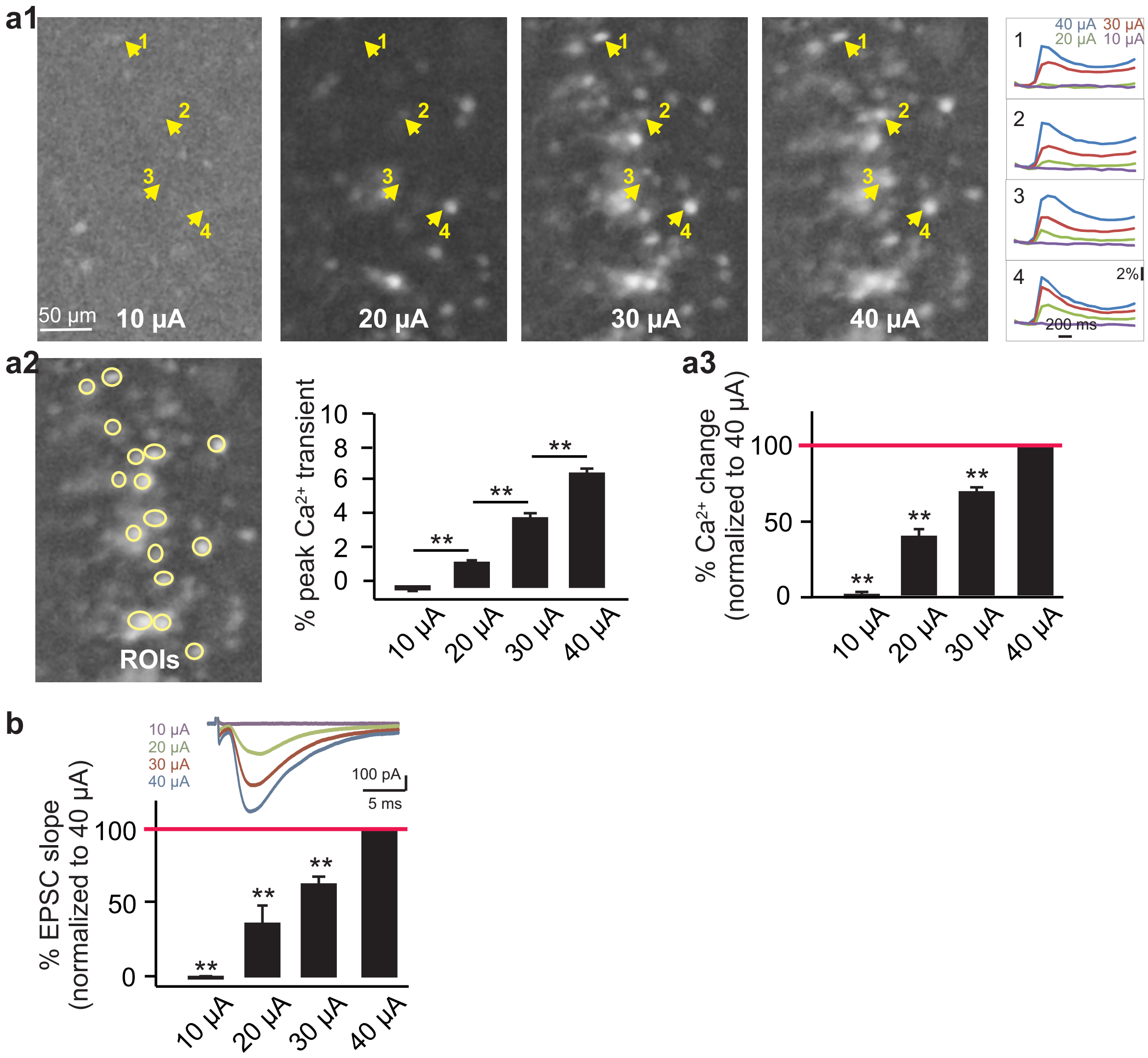

Relationship between calcium transients and stimulation intensities of the lateral olfactory tract (LOT)

(a1-a3) Somatic calcium transients show gradient increases with higher LOT stimulation intensities. (a1) Example images of population calcium imaging evoked by a single LOT stimulation at 10 µA, 20 µA, 30 µA and 40 µA. Images were constructed by averaging 4-6 frames of evoked calcium responses (ΔF/F) from 5 stimulus trials. Example calcium transient traces from 4 cells are shown on the right. (a2) Peak calcium transients (ΔF/F%) averaged from a population of cells (yellow circles) on the same slice. (a3) Normalized calcium changes (to control) during various LOT stimulations from 3 slices (n = 75 cells). (b) Percentage changes in EPSCs (normalized to 40 µA) evoked by various LOT stimulation intensities (n = 3).

EPSC recording method: Recordings of pyramidal cells in the aPC were performed with glass micropipettes (resistance 3-6 MΩ) pulled by a PC-10 puller (Narishige) and filled with a solution containing (in mM): 123 K-gluconate; 2 $MgCl_2$; 8 KCl; 0.2 EGTA; 10 HEPES; 4 Na_2-ATP; 0.3 Na-GTP). Cells were voltage clamped at -70 mV during whole-cell evoked EPSC recording. EPSCs were evoked by LOT stimulation. Average EPSCs of 15-20 sweeps recorded every 15 sec were used for data analysis and illustrations. Data acquisition employed a Multiclamp 700B amplifier (filtered at 2 kHz and digitized at 10 kHz), and pClamp10 software. Data was analyzed offline using Clampfit and Igor software.

Supplementary Figure 4

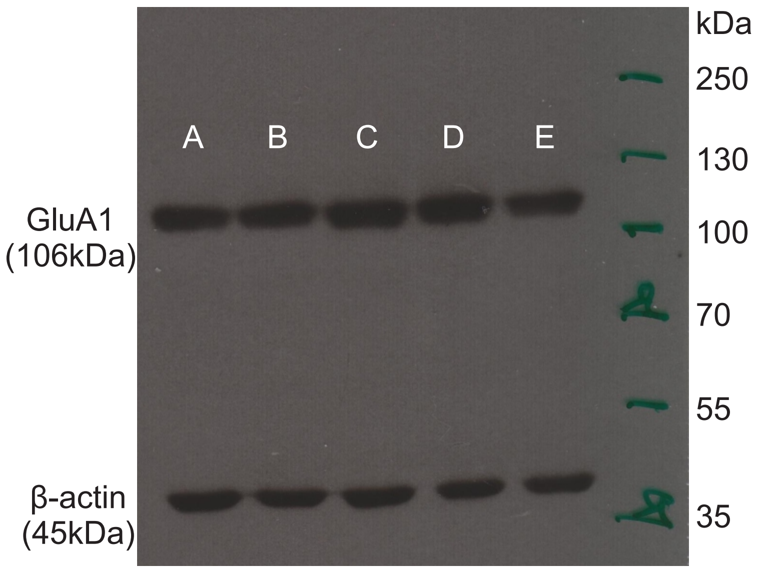

Full example blots for anterior piriform cortex AMPA receptor GluA1 expressions following various experimental conditions 3 hr post odor preference training

A. O/O+Vehicle; B. O/S+Vehicle; C. O/S+Nifedipine; D. O/S+Bay-K+Nifedipine; E. O/S+D-APV

Supplementary Figure 5

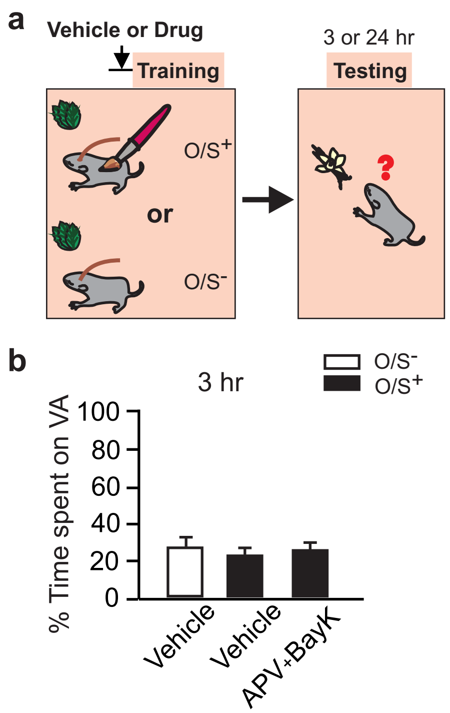

No generalized preference to a novel odor following L-type calcium channel activation

(a) Schematics of the odor preference training and testing paradigm. (b) Percentage of time spent over vanillin (VA)-scented bedding at 3 hr testing ($F_{2,9} = 0.72$, $p = 0.51$).
